# Health-first motivation as a demand-side strategy for pro-environmental consumption behavior: a cross-sectional retrospective online survey

**DOI:** 10.3389/fpubh.2026.1841511

**Published:** 2026-07-06

**Authors:** Yaeli Etstein, Adi Levi, Zohar Barnett-Itzhaki

**Affiliations:** 1 Ruppin Research Group in Environmental and Social Sustainability, Ruppin Academic Center, Emek Hefer, Israel; 2 Faculty of Marine Sciences, Ruppin Academic Center, Michmoret, Israel; 3 The Faculty of Health, Sciences and Sustainability, Achva Academic College, Yinon, Israel; 4 Faculty of Engineering, Ruppin Academic Center, Emek Hefer, Israel

**Keywords:** disposable equipment, environmental policy, health behavior, health policy, microplastics, social media, volatile organic compounds

## Abstract

**Introduction:**

Traditional environmental campaigns often struggle to achieve sustained behavioral shifts in reducing single-use products and their associated chemical exposure. This study examines whether sustained exposure to a health-centered social-media initiative, “Critical Mass for Healthy Consumption”, is associated with reported pro-environmental consumption patterns, by prioritizing personal health impacts over ecological concerns.

**Methods:**

Using an online survey, we compared consumer habits between community members (CM) and non-community members (NCM) across three categories: single-use cups for cold (SUCC) and hot (SUCH) beverages, and air fresheners.

**Results:**

CM reported significantly lower consumption of SUCC (11.4 cups/month, 95% CI: 10.5–12.3, *p* < 0.0001) compared to national estimates (16 cups/month). While environmental concern was high in both groups, CM rated personal health risks from chemical leaching and microplastics ingestion as their primary motivation for reduction. This divergence was most acute for air fresheners, a category lacking national environmental campaigns, where 39% of CM ceased use entirely compared to only 7% of NCM (difference: 32%, 95% CI: 23.5%–40.5%, *p* < 0.0001).

**Discussion:**

Our findings suggest that health-oriented communication may function as a stand-alone driver of behavior change in under-addressed product categories and that sustained health-oriented communication, enabled by digital community support and accessible alternatives, may act as an effective demand-side public health strategy to reduce chemical exposures and encourage pro-environmental behavior. Thereby, providing an empirical “Health-First” framework for integrating health-centered messaging, community-based platforms, and labeling schemes, into public health policy and education can promote safer, more sustainable consumption patterns.

## Introduction

Over the past two decades, global research has revealed an unprecedented rise in marine microplastics and nanoplastics (MP/NP) pollution. Short-lived single-use items are a substantial portion of this input, of which approximately 80% is transported via riverine systems (1). Beyond MP/NP pollution, single-use plastic cups impose a significant environmental footprint, generating greenhouse-gas emissions (2) and resource depletion through mismanaged plastic waste (3). The global air-freshener market, valued at $13.27 billion in 2022 is projected to reach $32.44 billion by 2032 (4). Air fresheners contribute to atmospheric pollution through both carbon emissions and the release of volatile organic compounds (VOCs). Hundreds of millions of single-use air-freshener containers are sold globally each year, with U.S. aerosol consumption alone exceeding four billion units annually (5) underscoring the dual burden of chemical emissions and packaging disposal. Household aerosols, constitute a substantial and growing source of urban organic emissions, while their short-lived packaging contributes disproportionately to resource-intensive waste streams (6, 7). Both single-use plasticware and air freshener also expose humans to hazardous chemicals, reflecting interconnected nature of environmental and public-health risks.

Plastic additives may migrate from single-use food-contact materials during routine use, a concern addressed in EU food-contact legislation (8). Plastic cups and packaging can release endocrine-disrupting additives, including phthalates, Bisphenols and per- and polyfluoroalkyl substances (PFAS), particularly when exposed to heat, acidic or oily media (9). Such compounds have been associated with reproductive toxicity, metabolic dysfunction and carcinogenic potential (10, 11). Disposable cups constitute a direct MP/NP ingestion route, with polyethylene-lined paper, polypropylene and polystyrene cups reported to release hundreds to several thousand particles per liter, with particle counts increasing with temperature and contact duration (12, 13). Growing scientific evidence indicates that MP/NP enter the human body and have been detected not only in human stool and blood (14), but also in the placenta (15), the liver (16), the kidneys (17), and the brain (18), further reinforcing health-motivated consumption shifts. Nevertheless, current exposure estimates should be interpreted with caution given ongoing methodological challenges in the MP/NP research field (19).

Air-fresheners emit VOCs, generating indoor concentrations of 30–160 μg/m^3^ (20). Chronic exposure to such emissions has been linked with asthma, allergies, migraines and endocrine disruption (21, 22). Beyond direct indoor exposure, VOC emissions from consumer products can participate in atmospheric reactions that generate ground-level ozone and secondary pollutants, further contributing to cardiometabolic and respiratory health risks (23–25). Collectively, this evidence suggests that everyday decisions about single-use products carry cumulative health consequences (10, 11, 21–23), framing these products as modifiable exposure sources within primary prevention.

Evidence that synthetic chemicals disrupt hormonal regulation in wildlife and humans (26) prompted advocacy organizations to develop science-based communication initiatives on chemical exposures from consumer products (27–29). Comparable consumer guidance has not been institutionalized in Israel. The “Critical Mass for Healthy Consumption” (CMHC) initiative was founded in 2016 to adapt this information to Israel's consumption landscape. Since its establishment, CMHC has continually adapted its health-oriented messages to the evolving scientific evidence on chemical and particulate exposures from everyday consumer products. A growing body of evidence indicates that health-oriented communication can be a powerful driver of health-protective and environmentally relevant behavior change, particularly when it increases perceived susceptibility to specific risks and highlights effective coping responses (30, 31).

In this study, we conceptualize CMHC as an application of Protection Motivation Theory (PMT), in which repeated, evidence-based information about microplastic, endocrine-disrupting chemical, and VOC exposures enhances threat appraisal (perceived severity and susceptibility), while the provision of feasible product alternatives and practical guidance strengthens coping appraisal (response efficacy and self-efficacy) (30, 32). Within PMT, such a combination of high perceived threat and high coping appraisal is expected to motivate adaptive responses, including the reduction or cessation of risky behaviors, rather than maladaptive coping responses (30, 31).

Building on prior PMT applications in health behavior and environmental health communication, we therefore characterize CMHC as a Health-First communication environment that mobilizes personal health concerns as a key motivational pathway for modifying everyday consumption practices. CMHC operates as a sustained communication environment engaging more than 16,000 individuals. CMHC is implemented as a Facebook-based community, which enables sustained, two-way interaction and community building at scale, while keeping the focus on health-oriented content and peer support rather than on the platform itself. It is characterized by: (i) a health-centered framing of chemical exposure risks provided by a trusted expert moderator, (ii) consistent pairing of risk information with feasible guidance toward safer alternatives, (iii) a scientific tone and structured explanations, and (iv) peer-to-peer reinforcement within a large, active social community. These features may together serve as a communication mechanism through which health risks are framed as directly relevant to personal and family wellbeing.

This framing may generate intrinsic motivation and support the generalization of health-oriented choices from one product domain to adjacent consumption categories, consistent with positive behavioral spillover, defined as the extension of change in one practice to related behaviors, particularly when reinforced through sustained communication (33). No comparable nationwide communication platform in Israel provided sustained, multi-category health-oriented consumer guidance combined with practical alternatives within a single, continuously maintained setting. Although several social media influencers addressed specific niches such as cosmetics, these sources were limited in scope.

Environmental campaigns or top-down regulations often trigger short-term reactions rather than lasting behavioral shifts, particularly when detached from people's immediate wellbeing (34, 35). This pattern has also been observed in national environmental campaigns in Israel. A national public awareness campaign targeting single-use plastics achieved broad visibility (36) but, despite two years of implementation, did not reverse rising consumption trends, reaching approximately 11 billion items annually (~1,200/capita) in 2020—among the highest rates worldwide (37). The 2021–2023 excise tax temporarily reduced sales by approximately 31–34%, but consumption quickly rebounded following its repeal (38, 39). In contrast, evidence indicates that framing environmental messages around personal health increases adoption of sustainable behaviors. Health-based motivations have been shown to reduce meat consumption (40), a pattern also reflected in the two decades-long, worldwide adoption of the Meatless Monday Movement's health-oriented dietary choices (41). Such effects may also extend beyond the initially targeted products. Research on behavioral spillover suggests that engagement with one pro-environmental or health-relevant practice may support related changes in adjacent domains (42), with positive spillover more likely when interventions target intrinsic motivation (33).

A systematic review found that information about hazardous chemicals in consumer products can affect preferences, product use, and product disposal, although these effects are highly context-dependent and vary by product type, consumer behavior, and the format of the information provided (43). Importantly, the review found that most available studies focus on household chemical products, where warning labels are already common, while less research has examined everyday consumer products in which the presence of hazardous chemicals is less apparent to consumers (43). This gap is particularly relevant to the product categories examined in the present study, where risks related to chemical and microplastic migration from single-use beverage cups and VOC emissions from air fresheners are not clearly communicated through product labels or consumer-safety frameworks.

As described above, CMHC addresses this gap by creating a communication mechanism that rapidly translates emerging health-relevant scientific findings into accessible consumer guidance and supports their uptake through practical alternatives and community reinforcement. Digital community platforms are particularly suited to this role. Social media has been shown to offer distinct advantages for health communication over traditional channels, including sustained peer interaction, accessible information sharing, and broader audience reach (44). This mechanism may have helped convert health concern into repeated purchasing decisions across adjacent consumer categories.

This study examines whether exposure to health information regarding chemical exposures from consumer products is associated with differences in self-reported use, reduction, cessation, and motivations. CM were treated as a higher-exposure group, repeatedly exposed to such information, whereas NCM lacked a comparable sustained communication environment. We focused on product categories: single-use cups for cold (SUCC) and hot (SUCH) beverages, and air fresheners. Single-use cups are high-consumption products that impose a substantial burden on Israel's waste-management system and have been addressed by national environmental campaigns with limited long-term success. SUCC and SUCH were analyzed separately because they differ in material composition, recyclability, and exposure context. Air fresheners were included as a contrasting category with no comparable history of national environmental campaigns in Israel and limited public access to health-related information on VOCs exposure. We hypothesized that greater information exposure would be associated with lower use or greater cessation, together with stronger health-related motivations for reduction.

## Methods

### Survey design and development

The survey was conducted online between August 15 and December 31, 2024, by researchers at the Ruppin Research Group in Environmental and Social Sustainability, Ruppin Academic Center, Israel. It employed a cross-sectional, retrospective assessment of self-reported changes in consumption behavior “before” and “after” exposure to health-oriented information about chemical and microplastic exposures from consumer products. The survey included multiple-choice questions, five-point Likert-scale questions, and open-ended questions, as detailed in the Supplementary material. We focused on three product categories with relevance to public health and environmental exposure: SUCC, SUCH, and air fresheners (see Supplementary material).

The questionnaire addressed key aspects such as frequency of use before and after a self-reported behavioral change attributed to exposure to scientifically validated health or environment-related information (hereafter, “the change”); the nature of this change (where and how it was made); motivations for the change; and barriers to adopting sustainable alternatives. In this context, “change” was defined as a self-reported decrease (reduction in frequency or quantity), cessation (complete discontinuation), or qualitative modification (substitution with an alternative product) in the use of SUCC, SUCH, or air fresheners attributed by the respondent to prior exposure to scientifically validated health or environment related information. These sub-types were treated as distinct categorical response options in the analysis, with an additional composite binary variable (“any change” vs. “no change”) used to standardize cross-group comparisons.

The questionnaire comprised five sections: (1) SUCC, (2) SUCH, (3) air fresheners, (4) general consumption habits covering additional product categories in which participants may have shifted to healthier alternatives, and (5) demographic and lifestyle characteristics, including gender and level of exposure to health-oriented information on harmful chemicals. Conditional display logic was applied so that product-specific follow-up questions within Sections 1–3 were shown only to participants who reported a behavioral change in that product category, thereby reducing respondent burden.

Since both exposure to information and consumption patterns were reconstructed retrospectively and measured at a single time point, the design limits causal inference, and the reported changes should be interpreted as perceived, self-attributed behavioral adjustments, rather than as definitive evidence of intervention effects.

The survey was administered online via Google Forms. Conditional display logic was applied so that product-specific follow-up questions were shown only to participants who reported a change in use for that product category, thereby reducing respondent burden and improving data quality.

### Survey distribution

The finalized survey was distributed online using three main routes, to maximize participant reach and diversity: (a) via the CMHC Facebook group (primary sample), conceptualized as a community-based digital health communication intervention; (b) via general Facebook groups; and **(c)** via WhatsApp groups, to increase demographic diversity.

Survey invitations were posted in the above-mentioned platforms and participants were invited to complete the questionnaire voluntarily.

To further motivate participation, a raffle was offered as an incentive for full survey completion. Informed consent was embedded within the survey's introductory section, which outlined the study's purpose and participation guidelines. Participants were assured of their anonymity, the confidentiality of their responses and their voluntary participation.

Community membership (CM) was determined based on self-reported affiliation with the CMHC community. Respondents who did not report affiliation with CMHC were categorized as non-community members (NCM). Group classification was based on a single self-reported item: respondents who indicated membership in the CMHC Facebook group were classified as CM; all others were classified as NCM. As this classification relied solely on self-reported, misclassification cannot be excluded, and findings should be interpreted accordingly.

### External data resources

Estimated data on yearly waste production for SUCC and SUCH were obtained from the Waste Treatment Division at the Israeli Ministry of Environmental Protection (MoEP) through official estimates provided via personal communication (personal communication, L. Goldberg, November 13, 2024).

These national estimates were used as benchmarking values for comparison with self-reported monthly consumption levels reported by survey participants.

### Data analysis

Survey's consistency and reliability was assessed using Cronbach's alpha for the following sections: the health motivation, the environmental motivation, and the behavioral change.

Monthly SUCC and SUCH consumption per person, reported by survey participants, was compared to MoEP estimates (16 SUCC and 7.5 SUCH cups per capita per month) using a one-sample *Z*-test. Ordinal frequency categories were converted to numeric monthly estimates using midpoint imputation, as follows: “never” = 0; “a few times per month (0–10)” = 5; “approximately once per day (11–30)” = 20; “3–4 times per day (31–60)” = 45; “more than 4 times per day (>120)” = 105.

Chi-square tests were used to compare categorical consumption patterns of SUCC, SUCH and air fresheners between CM and NCM, both before and after the change.

Non-parametric Wilcoxon rank-sum tests were used to compare motivations for reducing the consumption of SUCH, SUCC and air fresheners between CM and NCM, due to the ordinal nature of the Likert-scale data.

All statistical analyses were conducted using MATLAB^®^ version 2024b (MathWorks, Natick, MA, USA).

Statistical significance was defined as *p* < 0.05. Prior to analysis, responses containing logical inconsistencies or missing key variables were excluded.

For all between-group comparisons of motivation scores, the mean difference and its 95% confidence interval were calculated to complement the Wilcoxon rank-sum test *p*-value, using the standard error of the difference between two independent means.

### Ethical considerations

#### Participation was voluntary

All survey responses were fully anonymized prior to analysis; no personally identifiable information was retained in the analytic dataset. Anonymized data were stored in a password-protected folder accessible solely to the principal investigator and will be retained for 3 years following study completion, after which they will be permanently deleted.

## Results

In this work we report patterns of product use and cessation for SUCC, SUCH, and air fresheners, followed by motivations for change and cross-category shifts to healthier alternatives.

A total of 311 participants responded to the survey: 87.8% female and 12.2% male. The final analytic sample included 309 participants. Among CM respondents (*n* = 235), 41% reported visiting the group page at least once a week, whereas 6% reported visiting less than once a month. Seventy-three percent of CM respondents reported community membership exceeding 1 year.

Among NCM (*n* = 74), 74.7% reported no engagement with content related to chemical migration from consumer products into the human body.

Internal consistency of the health motivation scale (chemical leaching across four product categories) was assessed using Cronbach's alpha (α = 0.820), indicating good reliability. Similarly, the environmental motivation scale demonstrated good internal consistency (α = 0.824). The behavioral change scale showed acceptable-to-questionable reliability (α = 0.664).

The consumption of SUCC was estimated by the Israeli MoEP in 2021 at approximately 1.85 billion cups annually (personal communication, L. Goldberg, November 13, 2024). This corresponds to a mean of 195 SUCC cups per capita per year (approximately 16.3 cups per capita per month). Of note, the MoEP estimate is derived from administrative waste data rather than a population survey and reflects a different temporal window than the present study; it is therefore used here as the best available reference point for contextualizing consumption levels rather than as a precise inferential benchmark. In the present study, CM reported a mean consumption of 11.4 cups/month, significantly lower than the national estimate (95% CI: 10.5–12.3, *p* < 0.0001). NCM reported a mean consumption of 19 cups/month, which was not significantly different from the national estimate (*Z*-test, *p* = 0.4).

We then examined the consumption patterns before and after the change (i.e., exposure to health-related information regarding the use of single-use products). Across the entire sample, the change corresponded to a mean reduction of approximately three SUCC per month (77% decrease relative to pre-change mean consumption). More than 40% of respondents reported complete cessation of SUCC use, with similar rates of cessation among both CM and NCM. Reductions in SUCC consumption were statistically significant in both CM (*p* < 0.0001) and NCM (*p* = 0.027).

Comparison of SUCC consumption between the two groups (CM and NCM) showed that while there was no statistically significant difference between community and non-community responders regarding their SUCC consumption before the change (*p* = 0.1), there was such a difference after the change (*p* = 0.047). After the change, 58% of CM and 48% of NCM reported no SUCC consumption. Consumption of 1–10 SUCC per month was reported by 40% of CM and 44% of NCM, whereas consumption of >10 cups per month was reported by 1% and 8%, respectively. These findings indicate that after the change, 99% of the CM and 92% of the NCM reported either no SUCC consumption or only low-level consumption (1–10 cups per month).

**Figure 1:**
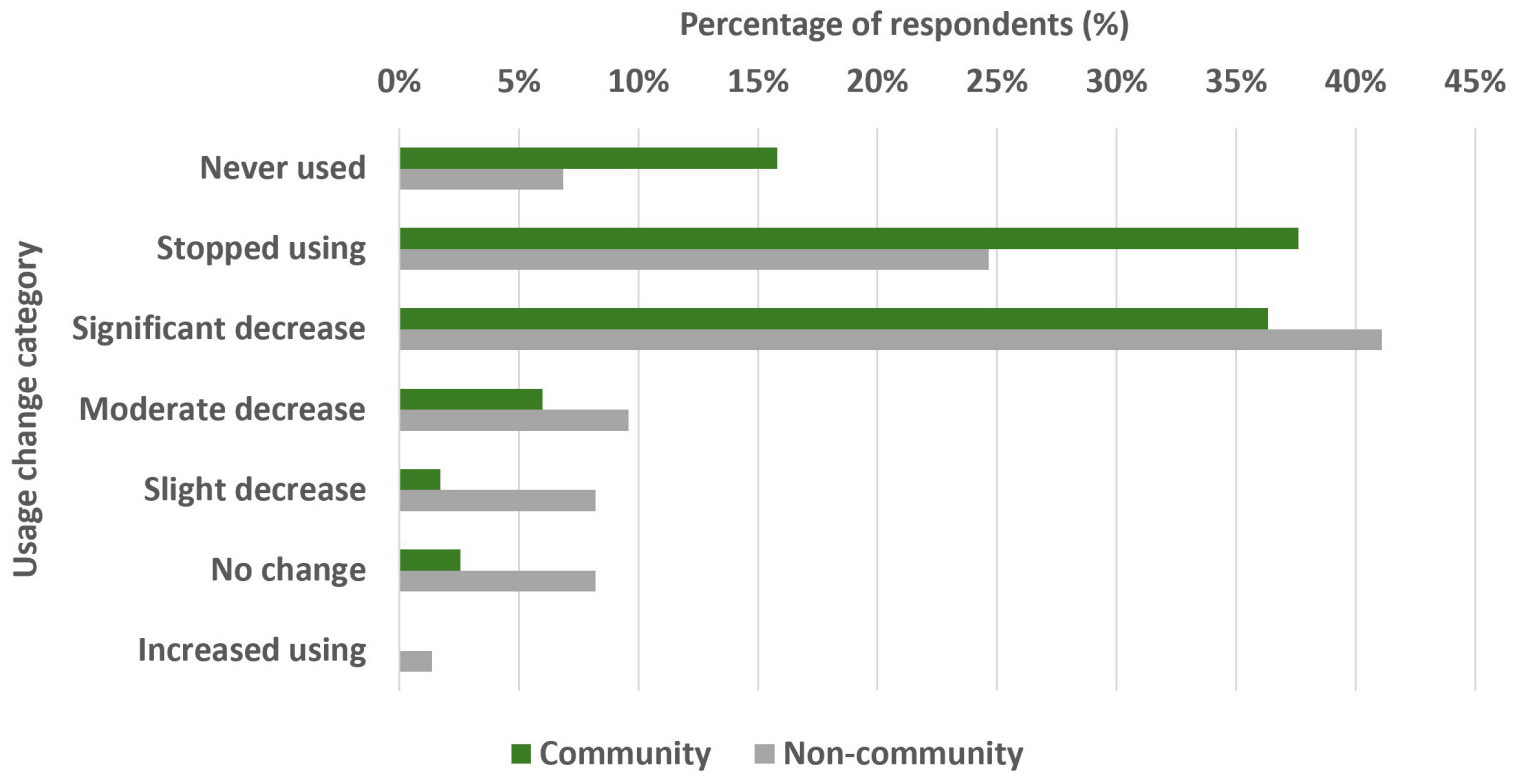
Self-reported changes in SUCC usage among CM and NCM (sample size: *N* = 309; CM: *n* = 235; NCM: *n* = 74).

Among CM, 18% had never used SUCC and 37.6% reported complete cessation following the change, compared with 6.8% and 24.7% among NCM, respectively (Figure 1).

Next, we examined the motivations for reducing SUCC consumption in both groups. This analysis was based on responses to questions regarding the reasons for reduction, where participants rated the relevance of each motivation on a five-point scale (1—not important to 5—very important). These comparisons include NCM respondents who did not report behavioral change and should therefore be interpreted with caution, as discussed in the discussion section.

Environmental pollution caused by single-use cups received high mean scores in both groups (CM = 4.60; NCM = 4.56; mean difference 0.04, 95% CI: −0.17–0.25, *p* = 0.59). Concern over the leaching of harmful chemicals and microplastic particles into the human body received higher mean scores among CM (4.48) compared to NCM (3.20; mean difference: 1.28, 95% CI: 0.91–1.66, *p* < 0.0001). Harm to wildlife due to cup waste received comparable mean scores (CM = 4.17; NCM = 4.08; mean difference 0.09, 95% CI: −0.22–0.4, *p* = 0.71). Reducing personal expenses received low mean scores in both groups (CM = 1.38; NCM = 1.69; *p* = 0.17; Table 1).

**Table 1:** Motivations for reducing consumption among CM and NCM (*n* = 309; CM: *n* = 235; NCM: *n* = 74).

Category	Motivation	CM^*^mean	NCM^*^mean	*p*-Value
SUCC^*^	Concern over the leaching of harmful chemicals and MP particles into the human body	**4.48**	**3.20**	**< 0.001**
	Environmental pollution	4.60	4.56	0.59
	Harm to wildlife due to cup waste	4.17	4.08	0.71
	Reducing personal expenses	1.38	1.69	0.17
SUCH^*^	Concern over the leaching of harmful chemicals and MP particles into the human body	**4.60**	**3.22**	**< 0.001**
	Environmental pollution	4.55	4.45	0.97
	Harm to wildlife due to cup waste	4.15	4.03	0.60
	Reducing personal expenses	1.47	1.71	0.29
	Awareness that cups are not recycled	3.69	3.52	0.61
Air fresheners	Concern over the leaching of harmful chemicals into the human body	**4.90**	**3.95**	**< 0.001**
	Environmental pollution	3.79	3.74	0.66
	Reducing personal expenses	1.42	2.05	0.08

Statistically significant differences (*p* < 0.05) are marked in bold.

^*^CM, community members; NCM, non-community members; SUCC, single use cups for cold beverages; SUCH, single use cups for hot beverages.

The consumption of SUCH was estimated by the Israeli MoEP in 2021 at approximately 850 million cups annually (personal communication, L. Goldberg, November 13, 2024). This corresponds to a mean of 90 SUCH per capita per year, or approximately 7.5 cups per capita per month.

We examined SUCH consumption before and after the change and observed a mean monthly reduction of approximately 11 cups (74% decrease relative to pre-change mean consumption). More than 40% of respondents reported complete cessation of SUCH use, with similar rates of cessation among both CM and NCM. After the change, 96% of CM and 85% of NCM reported either complete cessation or consumption of 1–10 SUCH per month. Reductions in SUCH consumption were statistically significant in both groups (CM: *p* < 0.0001, NCM: *p* = 0.021).

**Figure 2:**
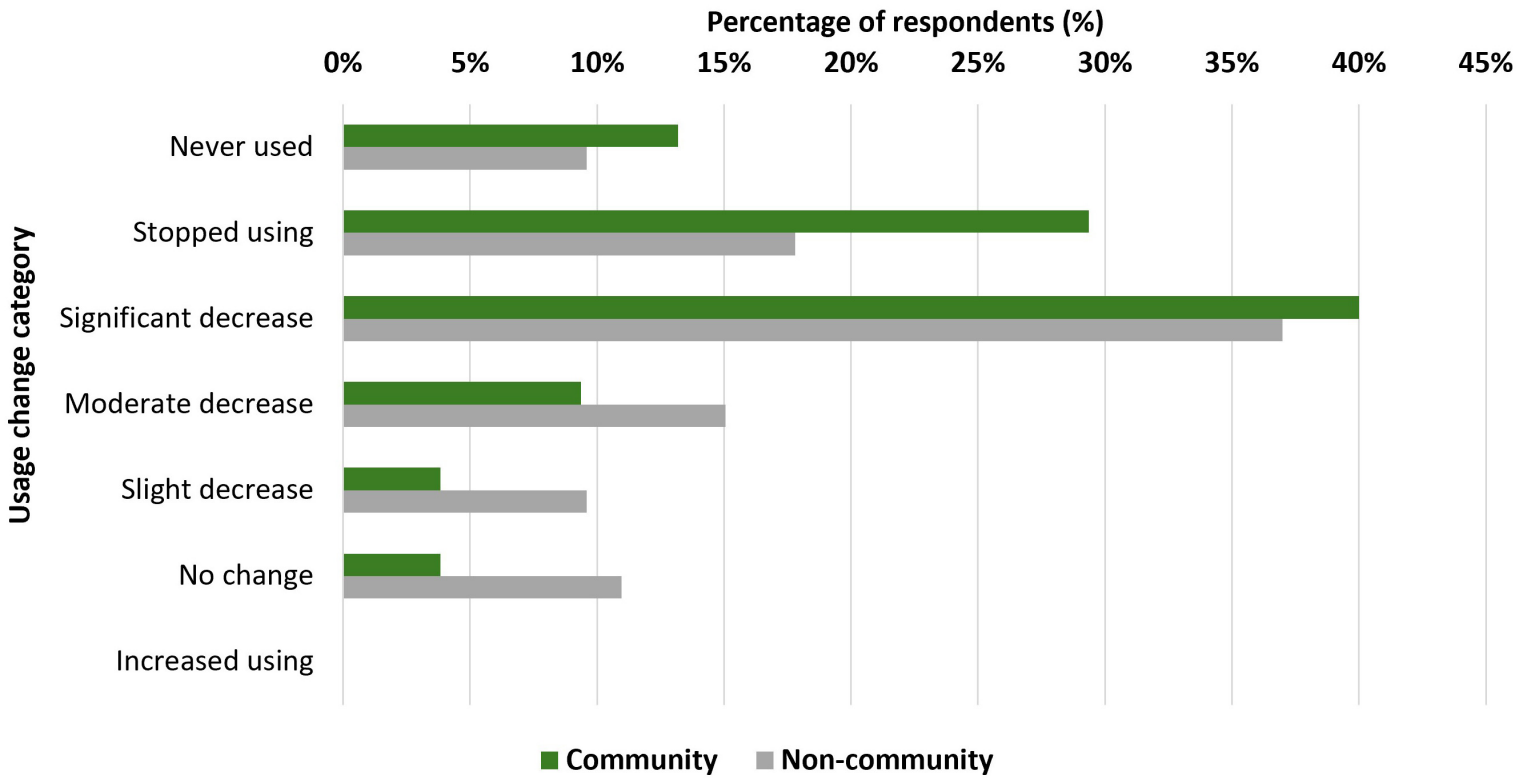
Self-reported changes in SUCH usage among CM and NCM (sample size: *n* = 309; CM: *n* = 235; NCM: *n* = 74).

Among CM, 13% reported never using SUCH and 29.4% reported complete cessation following the change, compared with 9.6% and 17.8% among NCM, respectively. Substantial reductions in SUCH use were reported by 40% of CM and 39% of NCM (Figure 2).

Comparison of SUCH consumption between groups showed statistically significant differences both before and after the change (*p* = 0.04, *p* = 0.003, respectively).

Next, we examined the motivations for reducing SUCH consumption in both groups. This analysis was based on responses to questions regarding the reasons for reduction, where participants rated the relevance of each motivation on a five-point scale.

Concern over the leaching of harmful chemicals and MP particles into the human body received higher mean scores among CM (CM = 4.60) than NCM (NCM = 3.22; mean difference: 1.38, 95% CI: 1.00–1.76, *p* < 0.0001). Environmental pollution caused by the cups received comparable mean scores in both groups (CM = 4.55; NCM = 4.45, mean difference: 0.1, 95% CI: −0.12–0.22, *p* = 0.97). Harm to wildlife due to cup waste also received comparable mean scores (CM = 4.15; NCM = 4.03; mean difference 0.12, 95% CI: −0.2–0.44, *p* = 0.60). Reducing personal expenses received low mean scores in both groups (CM = 1.47; NCM = 1.71; mean difference: −0.24, 95% CI: 0.71–0.23, *p* = 0.29; Table 1).

### Air fresheners use and reduction of VOC-related exposures

**Figure 3:**
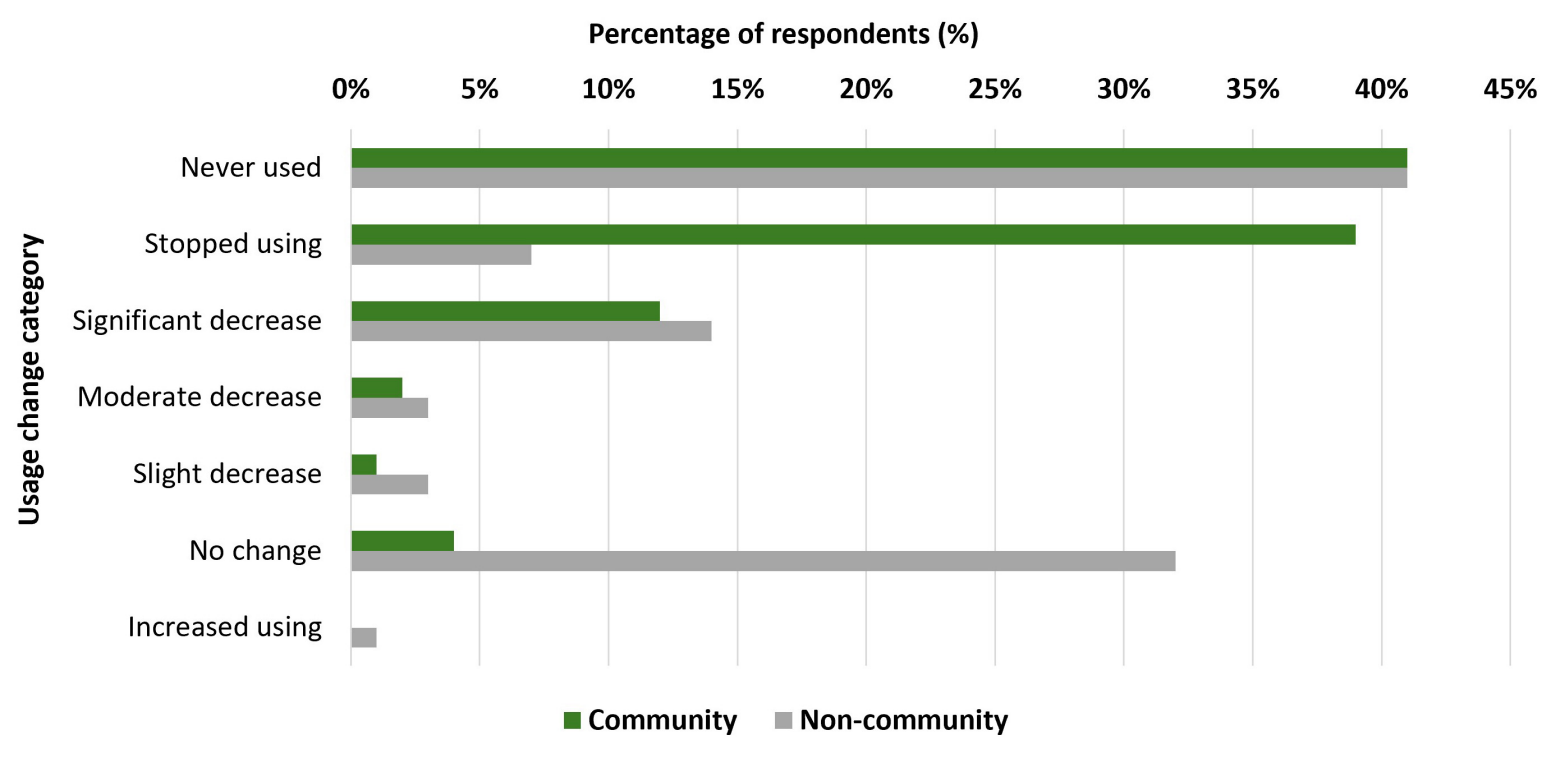
Self-reported changes in air fresheners usage among CM and NCM (sample size: *n* = 309; CM: *n* = 235; NCM: *n* = 74).

The central question regarding air freshener consumption was whether participants' usage had changed in recent years. Among both CM and NCM, 41% reported never having used air fresheners. 39% of CM reported complete cessation of air fresheners in recent years, compared to only 7% of NCM (difference: 32%, 95% CI: 23.5%–40.5%, *p* < 0.0001). 12% of CM and 14% of NCM reported a reduction in air freshener use. Four percent of CM and 32% of NCM reported no change in their usage over the years. The difference in air freshener usage trends between CM and NCM was statistically significant (*p* < 0.0001; Figure 3).

We next examined the motivations for reducing air freshener consumption in both groups. Concern over the leaching of harmful chemicals into the human body received higher mean scores among CM (CM = 4.90) than NCM (NCM = 3.95; mean difference: 0.95, 95% CI: 0.44–1.46, *p* < 0.001). Environmental pollution received comparable mean scores in both groups (CM = 3.79; NCM = 3.74; mean difference: 0.05, 95% CI: −0.54–0.64, *p* = 0.66). Reducing personal expenses received low scores in both groups (mean of CM = 1.42; NCM = 2.05, mean difference: −0.63, 95% CI: −1.27–0.01, *p* = 0.08; Table 1).

In the final part of the survey, participants were asked to report additional consumer product categories in which they had shifted to healthier alternatives. Differences between groups are presented in Table 2. Among CM, the most frequently reported categories were cosmetics and personal care (119 participants; 50.6%), cleaning products (101 participants; 43.0%), and cookware or kitchenware (94 participants; 40.0%). Smaller proportions reported changes in baby products (52 participants; 22.1%) and mattresses or sleep-related textiles (19 participants; 8.1%).

**Table 2:** Self-reported shifts to healthier alternatives across additional consumer product categories, based on an open-ended survey question, by group membership.

Consumer product category	CM^*^(*n*)	CM^*^(%)	NCM^*^(*n*)	NCM^*^(%)
Cleaning products	101	43.0	10	13.5
Cosmetics and personal care	119	50.6	9	12.2
Cookware and kitchenware	94	40.0	12	16.2
Baby products	52	22.1	6	8.1
Mattresses and bedding	19	8.1	2	2.7
No response	104	44.3	65	87.8

*n* = 309; CM: *n* = 235; NCM: *n* = 74.

^*^CM, community members; NCM, non-community members.

NCM reported lower frequencies across all categories, including cosmetics and personal care (9 participants; 12.2%), cleaning products (10 participants; 13.5%), cookware or kitchenware (12 participants; 16.2%), baby products (6 participants; 8.1%), and mattresses or sleep-related textiles (2 participants; 2.7%). Non-response to this item was more common among NCM (65 participants; 87.8%) than among CM (104 participants; 44.3%).

## Discussion

This study examined whether self-reported exposure to health-oriented information on chemical exposures from consumer products is associated with differences in consumption behavior and observed behavioral change.

CMHC members reported consistently lower baseline use, higher cessation rates (Figures 1–3), and broader cross-category adoption of healthier alternatives compared with non-members (Table 2), despite similarly high levels of environmental concern in both groups (Table 1). These findings are consistent with the hypothesis that health-oriented communication may support demand-side transitions toward more sustainable consumption.

SUCC and SUCH represent product categories that have been the focus of repeated national environmental campaigns in Israel (36, 45), driven by their substantial contribution to plastic pollution, marine litter and municipal waste loads (46–48). These campaigns emphasized ecological burden of single-use plastics but didn't include health-based messaging or practical guidance on reducing chemical migration or MP ingestion. Growing evidence shows disposable cups release substantial MP/NP particles during routine use (49) and represent a direct ingestion source for regular users (50). CM reported lower baseline SUCC and SUCH use than MoEP national estimates, whereas NCM patterns closely matched national averages (Figures 1, 2), consistent with the role of sustained health-oriented information and feasible substitution pathways.

CM described more pronounced reductions in SUCC and SUCH usage compared with NCM (Table 2), consistent with behavioral frameworks in which personal health relevance, availability of alternatives, peer modeling, and self-efficacy enhance motivation beyond environmental arguments alone (51, 52). SUCH consumption differed significantly between groups both before and after the change, suggesting that post-change differences may partly reflect pre-existing consumption patterns. Lower baseline consumption and higher cessation rates among CM suggest that health-oriented information may support the internalization and maintenance of pro-environmental consumption practices over time.

Air fresheners provide a contrasting case: unlike single-use cups, they have not been the focus of national environmental or chemical-risk campaigns in Israel. Nevertheless, their single-use packaging and cumulative contribution to VOC emissions from fragranced household products (22, 53), represent an under addressed demand-reduction target. CMHC has functioned as a persistent source of public awareness regarding the potential health implications of air freshener use, including respiratory irritants, endocrine-disrupting compounds, and indoor air quality concerns (22, 25). Accordingly, a far greater proportion of CM indicated that they had stopped using air fresheners (Figure 3) compared with NCM. Since both groups expressed similarly high levels of environmental concern (Table 1), and because no national campaigns have addressed this product category, the divergence may reflect differential access to sustained health-oriented explanations and feasible alternatives rather than differences in environmental attitudes alone.

The air freshener findings also illustrate a key feature of the CMHC communication model: its capacity to support informed, precautionary decision-making in the face of exposure variability. VOC emissions from consumer spray products vary substantially by product formulation, spray method, distance, and ventilation conditions (54), and air freshener compounds can react with airborne substances to generate secondary pollutants, while endocrine-disrupting fragrance solvents exhibit non-monotonic dose-response relationships (55), meaning that lower exposure does not necessarily correlate with proportionally lower risk. Rather than attempting to quantify safe usage parameters, CMHC fosters a precautionary and investigative approach—encouraging members to check product ingredients, assess personal sensitivity, and account for context-specific factors such as room size and ventilation. Consumption variability extends beyond individual households: in Israel, fragranced products are routinely deployed in retail stores, hotels, and healthcare facilities, frequently masking unpleasant odors rather than address their source, exposing individuals with respiratory conditions, chemical sensitivities, or hormonal vulnerabilities to unregulated compound mixtures. This raises a public health concern that transcends individual consumer choice and calls for institutional-level risk management. We therefore recommend that the CMHC precautionary health-centered approach be implemented in national health communication and policy.

A notably larger share of CM reported shifting to healthier alternatives across a range of everyday goods, including cleaning products, cosmetics, cookware, baby items and sleep-related textiles, compared with NCM (Table 2). This pattern suggests that individuals regularly engaging with sustained health-focused communication may generalize these principles to broader purchasing decisions, pointing to potential, theory–consistent spillover across consumer product categories (42). Substituting such items with lower-exposure or longer-lasting alternatives may also reduce demand for disposable materials, packaging intensity, and associated life cycle emissions. While not measured quantitatively, this cross-category shifts warrant further investigation.

Finally, beyond individual health concerns, the CMHC environment also embeds elements of peer support, including visible norms of low–exposure consumption, shared practical solutions, and ongoing exchange of experiences. Although our survey did not directly quantify peer–support processes, the co–occurrence of lower reported use, higher cessation rates, and broader cross–category shifts among CM is indicative of a social communication environment in which peer modeling and informational support reinforce the uptake and maintenance of healthier product choices, consistent with documented mechanisms in online sustainability communities (56). In the specific case of CMHC, expert-led guidance, practical substitution options, peer modeling within the continuous-feed format of a Facebook group may further support this process through repeated cross-category information, consistent with a Health-First process and positive behavioral spillover beyond the initially targeted products (33, 57).

### Study strengths and limitations

The strengths include: first, the examination of behavioral change within an ongoing, real-world, health-oriented consumer communication setting, thereby strengthening the public and environmental health relevance of the findings. Second, the inclusion of 309 valid responses and the direct comparison between CM and NCM across three product categories enabled a structured assessment of group-level behavioral differences. Third, combining reported consumption patterns with motivation ratings allowed behavioral outcomes to be interpreted alongside underlying motivations. Finally, comparison with Israeli MoEP consumption estimates provided a national benchmark for interpreting single-use cup consumption beyond the survey sample alone.

Several limitations of this study should also be acknowledged. First, group classification into CM and NCM was based on self-reported affiliation with the CMHC Facebook community, while engagement intensity was described but not analyzed as a dose-response predictor. Second, the cross-sectional, retrospective self-report design limits causal inference and did not include a baseline measure of health orientation before joining CMHC. Although CMHC administrative knowledge indicates that many members joined following exposure to CMHC content across other media platforms, this was not collected systematically for the 309 survey participants. Findings should be interpreted as associations, while pre-existing motivation cannot be formally ruled out but should not be assumed. Third, recall bias, social desirability bias, and motivation comparisons involving NCM respondents who did not report behavioral change may have affected reported consumption patterns and motivation ratings. Finally, the predominantly female sample, limited sociodemographic data, the specific Israeli policy and consumption context, and the focus on selected product categories may limit generalizability. Broader application to other settings and product domains should therefore be tested cautiously, although the observed cross-category patterns support further examination of health motivation as a potential driver of sustainable consumption spillover. The study also did not include objective consumption or exposure indicators, such as purchase records, product inventories, or biomarkers. To overcome these limitations, prospective longitudinal and quasi-experimental designs incorporating repeated measures of self-reported motivation alongside objective consumption indicators (such as purchase records, product inventories, or exposure biomarkers) are needed in larger, more diverse populations to establish causal relationships between health-oriented communication and sustained demand reduction. Future work should further examine how such communication can be integrated with regulatory, fiscal, and labeling frameworks, and whether positioning personal health protection as a central motivator promotes durable sustainable consumption shifts and reduces environmental footprints at scale.

### Policy implications

Our findings indicate that health-oriented communication, demonstrated by CMHC, can act as demand-side policy levers by reducing use of products linked to chemical exposures. Integrating these approaches into public health strategies could strengthen existing regulatory and fiscal measures by incorporating evidence-based risk communication, leveraging digital platforms for scalable engagement, and aligning policies with health-based labeling at the point of purchase. Expanding biomonitoring and consumption tracking would further support evaluation of these combined interventions, helping bridge the gap between scientific evidence and behavior.

## Conclusion

Everyday consumer products represent an important yet often overlooked source of chemical and microplastic exposure. This study, based on 309 survey responses, found statistically significant differences between CM and NCM in reported motivations to reduce SUCC, SUCH, and air freshener use due to concerns about chemical and MP exposure. CM reported larger reductions across the examined products, including notably high cessation rates for air fresheners, a category not addressed by national campaigns. Open-ended responses further suggested that health-based decision-making extended to additional product domains, indicating possible cross-domain diffusion of integrated health–sustainability practices. These findings support health-oriented consumer education, combined with accessible substitutes, as a promising demand-side public health strategy that may be more personally relevant than environmental messaging alone. However, the findings should be interpreted as associative rather than causal, and future studies should evaluate similar health-centered interventions across additional product categories and policy contexts.

## Data Availability

The raw data supporting the conclusions of this article will be made available by the authors, without undue reservation.
