# Supplementary material for: Health-first motivation as a demand-side strategy for pro-environmental consumption behavior: a cross-sectional retrospective online survey
Source: Front Public Health. 2026 Jul 6;14:1841511. doi: 10.3389/fpubh.2026.1841511 (PMC13381750; doi:10.3389/fpubh.2026.1841511)
Supplement: Supplementary file 1 [file Data_Sheet_1.docx]

**Supplementary material – Questionnaire**

**Changes in consumption habits following information on the release of chemicals from consumer products**

Hello everyone!
You are now taking part in a first-of-its-kind study that aims to characterize the impact of health-related information on the consumption of non-food consumer products —such as the information you may have encountered in lectures by Yaeli Etstein or through the content of the *Critical Mass* Facebook community.

What is this about?
We, researchers from Ruppin Research Group in Environmental and Social Sustainability, are interested in examining whether exposure to health-focused content, specifically information regarding the release of harmful chemicals from everyday consumer products and their effects on the human body, influences household consumption patterns.

We would like to understand whether any changes have occurred in your consumption of the following three product categories:

a. Disposable cups for cold drinks; b. Disposable cups for hot drinks; c. Air fresheners

**Why is this important?**
Every response helps us to better understand how to create more effective impact!

**What do you need to do?**

1. Read the questions carefully
2. Answer honestly, there are no right or wrong answers!
3. Complete the entire questionnaire: every response matters!

**A special bonus!**Everyone who completes the survey will be entered into a raffle for a valuable, non-toxic prize worth 1,000 NIS!

Thank you so much for your cooperation!
We wish you health and happiness,
Yaeli and the research team

**Disposable cups for cold drinks**

* Plastic cups used for drinking water, soft drinks, or alcohol, which are discarded after use. An illustrative image of disposable cups for cold beverages was included.

1. Has there been a change in your use of single-use cups for **cold** beverages in recent years? *(Multiple choice – single answer, ** *This response skips the remaining questions for this product category.)*

- I have never used disposable cups for cold drinks.*
- I have completely stopped using them (100% reduction).
- I have significantly reduced my use.
- I have moderately reduced my use (50% compared to the past).
- I have slightly reduced my use.
- No change has occurred.*
- I have increased my use.*

1. What was your monthly frequency of using single-use cups for **cold** beverages in the past, before the change in your consumption habits? *(Multiple choice – single answer)*

- A few times a month (0-10 cups per month)
- About once a day (11-30 cups per month)
- Once or twice a day (31-60 cups per month)
- Three to four times a day (90-120 cups per month)
- More than four times a day (More than 120 cups per month)

1. What is your current monthly frequency of using single-use cups for **cold** beverages, following the change in your consumption habits? *(Multiple choice – single answer)*

- I Do not use at all
- A few times a month (0-10 cups per month)
- About once a day (11-30 cups per month)
- Once or twice a day (31-60 cups per month)
- Three to four times a day (90-120 cups per month)
- More than four times a day (More than 120 cups per month)

1. To what extent was it easy or difficult to change your usage habits of single-use cups for **cold** beverages? *(5-point Likert scale: 1- very easy, 5- very hard)*
2. What are the main challenges you experience in trying to reduce the use of single-use cups for **cold** beverages? *(Open-ended)*
3. To what extent, if at all, did each of the following issues influence the change *in your consumption habits of single-use cups for* ***cold*** *beverages?
   (5-point Likert scale: 1- very little influence, 5- very big influence)*
   1. Leaching of harmful chemicals and microplastic particles from the cup into the human body.
   2. The environmental pollution that cups create.
   3. Harm to wildlife from cup waste.
   4. Desire to reduce my monthly expenses.
4. In which settings have your use of disposable cups for **cold** drinks decreased? *(Multiple answers allowed)*

- At home
- Stopped serving guests disposable cups
- At work (I have my own reusable cup)
- At restaurants and pubs (I ask for reusable cup)
- For takeaway (I have my own reusable cup)
- Traveling (I use a reusable kit or another alternative solution)
- At picnics (I use a reusable kit for events)
- I did not reduce

1. What did you choose to use instead of single-use cups for **cold** beverages?
   *(Multiple answers allowed)*

- Glass or ceramic cups
- Reusable plastic cup
- Reusable plastic water bottle
- Disposable plastic water bottle
- Stainless steel water bottle

**Disposable cups for hot drinks**

* Cups made of paper or cardboard used for drinking hot beverages and thrown away after use. An illustrative image of disposable cups for hot beverages was included.

1. Has there been a change in your use of single-use cups for hot beverages in recent years? *(Multiple choice – single answer, ** *This response skips the remaining questions for this product category.)*

- I have never used disposable cups for hot drinks.*
- I have completely stopped using them (100% reduction).
- I have significantly reduced my use.
- I have moderately reduced my use (50% compared to the past).
- I have slightly reduced my use.
- No change has occurred.*
- I have increased my use.*

1. What was your monthly frequency of using single-use cups for hot beverages in the past, before the change in your consumption habits? *(Multiple choice – single answer)*

- A few times a month (0-10 cups per month)
- About once a day (11-30 cups per month)
- Once or twice a day (31-60 cups per month)
- Three to four times a day (90-120 cups per month)
- More than four times a day (More than 120 cups per month)

1. What is your current monthly frequency of using single-use cups for hot beverages, following the change in your consumption habits? *(Multiple choice – single answer)*

- I Do not use at all
- A few times a month (0-10 cups per month)
- About once a day (11-30 cups per month)
- Once or twice a day (31-60 cups per month)
- Three to four times a day (90-120 cups per month)
- More than four times a day (More than 120 cups per month)

1. To what extent was it easy or difficult to change your usage habits of single-use cups for **hot** beverages? *(5-point Likert scale: 1- very easy, 5- very hard)*
2. What are the main challenges you experience in trying to reduce the use of single-use cups for **hot** beverages? *(Open-ended)*
3. To what extent, if at all, did each of the following issues influence the change *in your consumption habits of single-use cups for* **hot** *beverages? (5-point Likert scale: Did not influence, 1- very little influence, 5- very big influence)*
   1. Leaching of harmful chemicals and microplastic particles from the cup into the human body.
   2. The environmental pollution that cups create.
   3. Harm to wildlife from cup waste.
   4. Desire to reduce my monthly expenses.
4. In which settings have your use of disposable cups for **hot** drinks decreased? *(Multiple answers allowed)*

- At home
- Stopped serving guests disposable hot cups
- At work (I have my own reusable cup)
- At restaurants and pubs (I ask for reusable cup)
- For takeaway (I have my own reusable cup)
- Traveling (I use a reusable kit or another alternative solution)
- At picnics (I use a reusable kit for events)
- I did not reduce

1. What did you choose to use instead of single-use cups for **hot** beverages?  *(Multiple answers allowed)*

- Glass or ceramic cups
- Stainless steel cups
- Reusable plastic cups
- Reusable plastic water bottle
- Stainless steel water bottle

**Air Fresheners**

* *Important note - the questions in the following section refer to synthetic fragrances (petroleum- based). Regard all types of scenting products for spaces - scent sticks, automatic diffusers, sprays, and more*

1. Has there been a change in your use of air fresheners in recent years? *(Multiple choice – single answer, ** *This response skips the remaining questions for this product category.)*

- I have never used air fresheners.*
- I have completely stopped using them (100% reduction).
- I have significantly reduced my use.
- I have moderately reduced my use (50% compared to the past).
- I have slightly reduced my use.
- No change has occurred.*
- I have increased my use.*

1. How many synthetic air fresheners did you use in the past, before the change in your consumption habits? (The total number of products used you at home, in the car and at work). *(Multiple choice – single answer)*

- 1-2 units a month
- 3-5 units a month
- 6-10 units a month
- More than 10 units a month

1. How many synthetic air fresheners do you currently use, following the change in your consumption habits? (The total number of products used you at home, in the car and at work). *(Multiple choice – single answer)*

- I Do not use at all now
- 1-2 units a month
- 3-5 units a month
- 6-10 units a month
- More than 10 units a month

1. To what extent was it easy or difficult to change your usage habits of synthetic air fresheners? *(5-point Likert scale: 1- very easy, 5- very hard)*
2. What are the main challenges you experienced in trying to reduce the use of synthetic air fresheners? *(Open-ended)*
3. To what extent, if at all, did each of the following issues influence the change in your consumption habits of synthetic air fresheners? *(5-point Likert scale: Did not influence, 1- very little influence, 5- very big influence)*
   1. Dispersal of chemicals that are harmful to the human body.
   2. The environmental pollution that this industry creates.
   3. Desire to reduce my monthly expenses.
4. Where is the reduction in use of synthetic air fresheners expressed? *(Multiple answers allowed)*

- At home
- In the car
- At work/in my business

1. What did you choose to use instead of synthetic air fresheners? *(Multiple answers allowed)*

- Burner or electric diffuser for essential oils
- Incense
- Natural compound air fresheners (scent sticks, scent sponges)
- Another type of DIY air freshener
- I stopped using air fresheners and did not replace them with another product

**General questions**

1. Are there other consumer products in which your consumption habits changed as a result of receiving information about potentially harmful chemical leaching from these products?

- Natural cosmetic products
- Cleaning materials
- Cooking utensils
- Sleeping mattresses
- Baby products
- Other

1. You can write here about any change you made in these products or mention other consumer products in which you made a significant change as a result of receiving information about potentially harmful chemicals leaching.
   *(Open-ended)*
2. Gender (M/F/Refuse to answer)
3. When did you join Critical Mass? *(Multiple choice – single answer)*

- I am not a member of the "Critical Mass" group
- In the last month
- 1–6 months ago
- More than six months ago
- More than a year ago
- I have already been in the group for several years

How frequently do you visit the group? *(Multiple choice – single answer)*

- I am not a member of the group
- Very frequently (4–5 times a week)
- Frequently (1–3 times a week)
- Sometimes (once every two to three weeks)
- Rarely (once a month)
- Very rarely
- Never

1. Do you consume content about migration of harmful chemicals from consumer products into the human body from sources other than Critical Mass? If answered yes - who are those sources? *(Open-ended)*

Thank you for answering!

For the benefit of the raffle, please provide your full name and contact email
